# Advancements in Antibacterial Therapy: Feature Papers

**DOI:** 10.3390/microorganisms13030557

**Published:** 2025-03-01

**Authors:** Giancarlo Angeles Flores, Gaia Cusumano, Roberto Venanzoni, Paola Angelini

**Affiliations:** 1 Department of Chemistry, Biology and Biotechnology, University of Perugia, Via del Giochetto, 06122 Perugia, Italy; giancarlo.angelesflores@unipg.it (G.A.F.); gaia.cusumano@dottorandi.unipg.it (G.C.); roberto.venanzoni@unipg.it (R.V.); 2 Centro di Ricerca per l’Innovazione, Digitalizzazione, Valorizzazione e Fruizione del Patrimonio Culturale e Ambientale (CE.D.I.PA.), Piazza San Gabriele dell’Addolorata, 4, 06049 Spoleto, Italy

**Keywords:** antimicrobial peptides (AMPs), bacteriophage, biofilm, broad-spectrum antibiotics, CRISPR-based antimicrobials, extracellular vesicles (EVs), MDR bacteria, nanotechnology, vaccines

## Abstract

Antimicrobial resistance (AMR) is a growing global health crisis that threatens the efficacy of antibiotics and modern medical interventions. The emergence of multidrug-resistant (MDR) pathogens, exacerbated by the misuse of antibiotics in healthcare and agriculture, underscores the urgent need for innovative solutions. (1) Background: AMR arises from complex interactions between human, animal, and environmental health, further aggravated by the overuse and inadequate regulation of antibiotics. Conventional treatments are increasingly ineffective, necessitating alternative strategies. Emerging approaches, including bacteriophage therapy, antimicrobial peptides (AMPs), nanotechnology, microbial extracellular vesicles (EVs), and CRISPR-based antimicrobials, provide novel mechanisms that complement traditional antibiotics in combating resistant pathogens. (2) Methods: This review critically analyzes advanced antibacterial strategies in conjunction with systemic reforms such as antimicrobial stewardship programs, the One Health framework, and advanced surveillance tools. These methods can enhance resistance detection, guide interventions, and promote sustainable practices. Additionally, economic, logistical, and regulatory challenges impeding their implementation are evaluated. (3) Results: Emerging technologies, such as CRISPR and nanotechnology, exhibit promising potential in targeting resistance mechanisms. However, disparities in resource distribution and regulatory barriers hinder widespread adoption. Public–private partnerships and sustainable agriculture practices are critical to overcoming these obstacles. (4) Conclusions: A holistic and integrated approach is essential for mitigating the impact of AMR. By aligning innovative therapeutic strategies with global health policies, fostering interdisciplinary collaboration, and ensuring equitable resource distribution, we can develop a sustainable response to this 21st-century challenge.

## 1. Introduction

Antimicrobial resistance (AMR) represents a major global healthcare crisis, threatening the effectiveness of treatments against infectious diseases [1]. The proliferation of multidrug-resistant (MDR) pathogens, including *Staphylococcus aureus*, *Escherichia coli*, and *Klebsiella pneumoniae*, has rendered many previously treatable infections increasingly persistent [2]. Factors such as the misuse of broad-spectrum antibiotics, non-adherence to prescribed treatments, and excessive antibiotic use in agriculture have accelerated the spread of resistance, facilitating the transmission of resistant strains across human, animal, and environmental ecosystems [3]. Compounded by a stagnation in antibiotic discovery, this crisis necessitates urgent and innovative solutions [4].

AMR fundamentally undermines treatment efficacy, as bacteria rapidly evolve to evade antibiotics, posing severe risks to public health [5]. While antibiotics have historically revolutionized medicine by reducing mortality and enabling complex surgeries, their indiscriminate use has driven the evolution of resistant pathogens, rendering many treatments ineffective [6]. The lack of new antibiotic classes exacerbates this challenge, emphasizing the need for alternative therapies [7]. Efforts to combat AMR must address its root causes while fostering innovation. Promising alternatives include antimicrobial peptides, bacteriophage therapy, immune system modulators, and small-molecule antibiotics capable of bypassing traditional resistance mechanisms [4,5,6,7,8]. However, progress is hindered by regulatory barriers, high development costs, and lengthy approval processes [6]. Given AMR’s interconnected nature across human, animal, and environmental health, a One Health approach is imperative. The agricultural misuse of antibiotics, particularly as growth promoters in livestock, significantly contributes to resistance proliferation. Resistant strains can enter food systems and natural ecosystems, perpetuating the cycle [1]. Addressing this issue necessitates stringent regulatory frameworks, increased public awareness, and the adoption of sustainable alternatives such as probiotics and vaccines [7]. Surveillance systems play a critical role in monitoring resistance trends and informing public health policies. Advances in molecular diagnostics, predictive modeling, and artificial intelligence enhance the early detection and containment of resistant strains [7,8]. Global cooperation and harmonized regulations are essential to mount an effective response to AMR. A fundamental shift in antibiotic development, distribution, and usage is required, encompassing increased investment in research and development, incentives for pharmaceutical innovation, and public–private partnerships to bridge gaps in discovery and deployment [8]. Furthermore, global antimicrobial stewardship programs must be strengthened to optimize antibiotic use and prevent unnecessary prescriptions [9].

In summary, AMR is an escalating global health crisis with profound implications. By integrating cutting-edge therapeutic innovations, robust regulatory policies, and a One Health approach, the global community can effectively combat AMR, preserve the efficacy of existing antibiotics, and safeguard future generations from untreatable infections.

## 2. Materials and Methods

**Figure 1:**
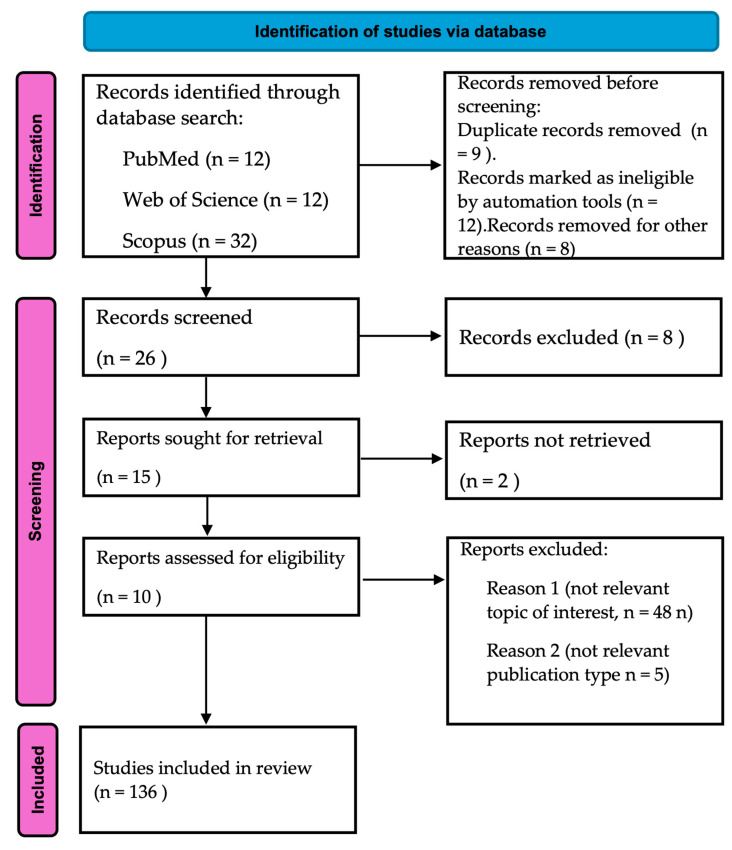
Study flowchart according to the PRISMA recommendations [10]. The diagram depicts the sequential stages of identification, screening, eligibility assessment, and final inclusion of studies. It provides a detailed overview of the number of records retrieved, excluded, and ultimately incorporated into the systematic review.

This review systematically examines advancements and challenges in addressing antimicrobial resistance (AMR), focusing on innovative antibacterial strategies and their integration into broader healthcare frameworks. Relevant research from 2000 to 2025 was retrieved from databases such as PubMed (National Library of Medicine, Bethesda, MD, USA), Scopus (Elsevier, Amsterdam, The Netherlands), and Web of Science (Clarivate, Philadelphia, PA, USA) using search terms including “antimicrobial resistance”, “multidrug-resistant pathogens”, “bacteriophage therapy”, “antimicrobial peptides”, “nanotechnology in antimicrobials”, “microbial extracellular vesicles”, and “CRISPR-based antimicrobials”. Studies investigating novel therapeutic approaches, AMR mechanisms, and policy frameworks were selected for inclusion. This review evaluates the mechanisms, efficacy, and limitations of emerging strategies, including bacteriophage therapy, antimicrobial peptides, nanotechnology, microbial extracellular vesicles, and CRISPR technologies, in conjunction with traditional antibiotics. Additionally, systemic approaches such as antimicrobial stewardship programs, the One Health framework, and advanced surveillance tools—particularly those incorporating artificial intelligence (AI)—were analyzed. Key economic, regulatory, and interdisciplinary barriers were identified and discussed to highlight both challenges and opportunities for global implementation. Since this study is based on secondary data from peer-reviewed sources, ethical approval was not required. This review adheres to standardized reporting guidelines and follows the PRISMA 2020 (Preferred Reporting Items for Systematic Reviews and Meta-Analyses) framework [10]. The PRISMA 2020 checklist was employed to assess methodological rigor and reporting compliance. Figure 1 illustrates the content distribution map for this review, providing an overview of the study selection process.

Table 1 outlines the essential reporting items, their descriptions, and the extent to which they were addressed in this study, ensuring transparency and adherence to best practices.

## 3. The Global Threat of Antibiotic Overuse and Misuse

The overuse and misuse of antibiotics pose a severe and escalating threat to global health, fueling the rise in antimicrobial resistance (AMR) and undermining the efficacy of antibiotics that are essential for treating infections and sustaining modern medicine. The complexity of AMR demands a comprehensive and multifaceted approach that not only addresses its root causes in both medical and agricultural settings but also fosters the development of innovative solutions to counteract its progression [11]. In healthcare, inappropriate antibiotic prescriptions, particularly for viral infections, along with the premature discontinuation of treatments by patients, contribute significantly to the emergence of resistant bacterial strains. Additionally, limited access to accurate diagnostics and widespread misconceptions about antibiotic use among both healthcare providers and the general public further exacerbate the issue, leading to the unnecessary and excessive use of antimicrobial agents [12].

Beyond clinical settings, agricultural practices also play a substantial role in the acceleration of AMR. The widespread administration of antibiotics in livestock farming for therapeutic purposes, growth promotion, and prophylaxis has created an environment of intense selective pressure, fostering the emergence and proliferation of resistant bacterial strains. These resistant pathogens can reach humans through the consumption of contaminated food, direct animal contact, and environmental pathways such as water runoff and soil contamination, particularly in regions where regulatory oversight is weak or inconsistent [13,14]. The risks associated with agricultural antibiotic use are especially pronounced in low- and middle-income countries, where inadequate regulatory frameworks and poor enforcement facilitate the bidirectional transmission of resistant bacteria between human and animal ecosystems, making containment increasingly challenging [15,16].

Effectively combating AMR requires a One Health approach that acknowledges the interconnectedness of human, animal, and environmental health. Global mobility, inadequate sanitation, and overcrowding contribute to the rapid dissemination of resistant pathogens, highlighting the urgent need for cross-sector collaboration to control and contain the crisis. The implementation of antimicrobial stewardship programs (ASPs) in healthcare settings is crucial to optimizing antibiotic use, improving diagnostic capabilities, enforcing treatment guidelines, and strengthening infection control measures. Concurrently, public education campaigns play a vital role in raising awareness and promoting responsible antibiotic use, ensuring that both healthcare professionals and the general population are equipped with the necessary knowledge to mitigate resistance risks [17,18].

In the agricultural sector, reducing antibiotic use, particularly as growth promoters, is a critical step toward addressing AMR. Sustainable alternatives such as improved hygiene, targeted vaccination, and the use of probiotics offer viable solutions for maintaining livestock productivity without exacerbating resistance. However, these measures must be reinforced by stronger international regulations and enhanced oversight mechanisms to ensure the responsible use of antibiotics and prevent the uncontrolled spread of resistance between human and animal populations [19,20].

Compounding the crisis is the stagnation in antibiotic development, which further exacerbates the AMR burden and underscores the urgent need for renewed investment in research and innovation. The exploration of alternative therapeutic strategies, including plant-derived antimicrobials, bacteriophage therapy, and synthetic biology-based antimicrobial agents, presents promising avenues for addressing the limitations of conventional antibiotics. Additionally, expanding global surveillance systems to monitor resistance trends, track emerging threats, and standardize international regulatory efforts is essential for mounting a coordinated global response and ensuring the effectiveness of both existing and novel antimicrobial interventions [21,22].

Antimicrobial resistance represents an urgent and growing global health crisis that demands immediate and coordinated action. By integrating antimicrobial stewardship initiatives, implementing agricultural reforms, and advancing cutting-edge research within the One Health framework, the global community can take meaningful steps toward mitigating the devastating impact of AMR. Ensuring the responsible use of antibiotics, reinforcing regulatory policies, and fostering scientific innovation will be crucial in preserving the efficacy of antimicrobial treatments and safeguarding public health for future generations. Without decisive action, the world risks facing a future where routine infections become increasingly difficult to treat, posing a profound threat to modern medicine and global healthcare infrastructure.

## 4. Antibiotic Use in Agriculture: A Catalyst for Resistance and Strategies for Mitigation

The widespread use of antibiotics as growth promoters in livestock has significantly accelerated the spread of antimicrobial resistance (AMR) through the food chain [23]. Administered subtherapeutically to prevent disease and enhance growth, antibiotics create strong selective pressure, facilitating the proliferation of resistant bacterial strains within animal populations [24,25]. These resistant bacteria can reach humans through direct contact with livestock, the consumption of contaminated food, and environmental pathways such as agricultural water runoff and soil contamination [26]. The extensive use of antibiotics in agriculture not only affects livestock but also alters commensal and environmental microbiota, contributing to the dissemination of resistance genes across microbial ecosystems. This process leads to the formation of resistance reservoirs in soils, water bodies, and other environmental niches, playing a pivotal role in the cross-species transmission of resistance within the One Health framework [26,27].

The absence of standardized global regulations exacerbates the problem. In many regions, antibiotics remain readily available over the counter, leading to unregulated and indiscriminate use. Weak surveillance systems and ineffective enforcement mechanisms further hinder efforts to monitor and control resistance development [28]. Addressing these challenges requires the implementation of strict regulatory policies to limit the non-therapeutic use of antibiotics in agriculture, stronger antimicrobial stewardship programs to promote responsible antibiotic practices, and robust monitoring systems to track antibiotic residues and resistance patterns in both agricultural and environmental contexts [28]. Without these measures, the continuous spread of resistance through agricultural practices poses a significant threat to human and animal health, as well as to overall ecosystem stability.

The adoption of sustainable alternatives to antibiotics in livestock production offers a viable solution to mitigating resistance risks. The use of probiotics, prebiotics, and vaccines has shown great potential in improving animal health and productivity while minimizing the selective pressure that drives antimicrobial resistance. Probiotics enhance gut microbiota, promoting better nutrient absorption and immune function, while vaccines target specific pathogens, reducing the necessity for prophylactic antibiotic use [29,30]. These strategies align with sustainable agricultural principles and provide an effective means of maintaining livestock productivity without compromising public health.

A comprehensive approach that integrates regulatory frameworks, innovative alternatives, and enhanced surveillance within the One Health paradigm is essential to combating antimicrobial resistance. This holistic strategy recognizes the interconnected dynamics of human, animal, and environmental health, underscoring the need for coordinated efforts to curb resistance and protect future generations. By implementing stricter policies, promoting sustainable agricultural practices, and investing in advanced monitoring and surveillance, the global community can mitigate the impact of AMR while ensuring food security and environmental sustainability.

## 5. The Decline in Antibiotic Development: Challenges and Proposed Solutions

The decline in antibiotic development presents a major global health challenge, primarily driven by economic disincentives and structural barriers within the pharmaceutical industry [31]. In recent decades, pharmaceutical companies have increasingly prioritized more profitable therapeutic areas such as oncology and chronic diseases, as antibiotics—typically prescribed for short durations—generate significantly lower revenues than long-term treatments [32,33]. This shift in investment is particularly alarming given the continuous rise in antimicrobial resistance (AMR), which is eroding the efficacy of existing antibiotics and threatening global health advancements.

Several economic and regulatory barriers further discourage antibiotic innovation. New antibiotics are often reserved for severe or drug-resistant infections to delay resistance development, which significantly reduces their market potential and profitability [34]. Additionally, stringent regulatory requirements—including extensive safety evaluations, efficacy assessments, and post-market surveillance—substantially increase development costs and prolong approval timelines, disproportionately affecting smaller biotechnology firms that lack the financial resilience to sustain these lengthy and costly processes [35]. These challenges have led to a stagnation in antibiotic discovery, exacerbating the already critical AMR crisis and reducing the pipeline of novel antimicrobial agents.

Addressing these barriers requires a fundamental restructuring of financial incentives and regulatory frameworks to stimulate antibiotic research and development. Public–private partnerships (PPPs) have emerged as a crucial mechanism for risk-sharing and resource pooling, allowing early-stage research and development to advance while mitigating financial burdens on pharmaceutical companies. Market entry rewards offer additional incentives by providing financial benefits upon the successful launch of new antibiotics, counteracting the historically low profitability of antimicrobial drugs [31]. Furthermore, targeted grant programs, such as CARB-X and GARDP, have been instrumental in prioritizing funding for research into drug-resistant pathogens, effectively supporting academic institutions and smaller biotechnology firms that are at the forefront of antibiotic discovery [36]. Strengthening collaboration between academia and the pharmaceutical industry is equally essential in revitalizing antibiotic development. Academic research institutions play a crucial role in identifying novel antimicrobial mechanisms and therapeutic targets, yet these discoveries often require industry partnerships to progress into clinically viable treatments. Advances in genomics, synthetic biology, and precision medicine have further enhanced the potential for developing next-generation antibiotics tailored to combat emerging resistance patterns, but translating these innovations into market-ready drugs necessitates stronger cooperation between research institutions and pharmaceutical enterprises [37]. In addition to revitalizing antibiotic development, ensuring the sustainable use of existing antibiotics is paramount. Antimicrobial stewardship programs (ASPs) play a critical role in promoting responsible prescribing practices to prolong the efficacy of both current and future antibiotics. The implementation of combination therapies and adjunctive treatments, which pair antibiotics with synergistic compounds, has also been shown to enhance treatment effectiveness while reducing the risk of resistance development [38]. These strategies represent vital components of a multifaceted approach to preserving antibiotic efficacy and mitigating the AMR crisis.

Given the urgency of the situation, coordinated global action is required to address the antibiotic development crisis. Governments, healthcare systems, academia, and the private sector must collaborate to implement key policy measures that encourage antibiotic innovation. Tax incentives and advanced purchase commitments can provide financial motivation for pharmaceutical companies to reinvest in antibiotic research and development. Streamlining regulatory processes can help accelerate the approval of new antibiotics while maintaining rigorous safety and efficacy standards. Increased funding and international collaboration will be crucial to strengthening the global antibiotic pipeline and ensuring that new treatments continue to reach the market [39].

Without decisive intervention, the world risks entering a post-antibiotic era in which routine infections could once again become life-threatening, fundamentally undermining modern medical advancements. The urgency of the issue cannot be overstated, and immediate, collective action is essential to safeguard public health and ensure a sustainable future for antibiotic innovation. A coordinated effort to overcome the economic, regulatory, and scientific challenges surrounding antibiotic development is necessary to curb the AMR crisis and preserve the effectiveness of antimicrobial therapies for future generations.

## 6. Unveiling the Complex Mechanisms of Antibiotic Resistance: Challenges and Prospects for Novel Therapeutics

Bacterial pathogens exhibit exceptional adaptability under antibiotic pressure, employing a variety of resistance mechanisms that pose significant challenges to modern medicine. One of the most well-documented strategies is enzymatic degradation, in which bacteria produce hydrolytic enzymes such as β-lactamases, which cleave the β-lactam ring of antibiotics, rendering penicillins, cephalosporins, and carbapenems ineffective. The structural diversity of β-lactamases, as emphasized by Rajput et al. [40], complicates treatment strategies and reduces the efficacy of even advanced β-lactam-based therapies.

Another key resistance mechanism involves efflux pumps, which actively expel antibiotics from bacterial cells, lowering intracellular drug concentrations to subtherapeutic levels. The overexpression of these pumps not only decreases antibiotic susceptibility but also enhances bacterial virulence, making infections significantly more difficult to treat. As highlighted by Gaurav et al. [41], the development of efflux pump inhibitors is critical for restoring drug efficacy and improving treatment outcomes.

Biofilm formation presents an additional challenge, particularly in chronic infections and those associated with medical devices. The extracellular polymeric substance (EPS) matrix surrounding bacterial biofilms serves as a physical barrier, impeding antibiotic penetration, facilitating bacterial communication through quorum sensing, and promoting a metabolically dormant state, which enhances bacterial tolerance to antimicrobial agents. Sharma et al. [42] report that biofilm-associated infections exhibit significantly higher resistance to conventional treatments, often necessitating combination therapies or the use of novel antibiofilm agents for successful eradication.

Another widespread resistance mechanism is target modification, which allows bacteria to evade antibiotics by altering key drug-binding sites such as ribosomal domains, DNA gyrase, and penicillin-binding proteins (PBPs). These structural modifications prevent effective drug–target interactions, rendering antibiotics such as fluoroquinolones, oxazolidinones, and β-lactams ineffective. Brdová et al. [43] describe how methicillin-resistant *Staphylococcus aureus* (MRSA) acquires resistance by expressing PBP2a, a low-affinity penicillin-binding protein, which prevents β-lactam antibiotics from effectively inhibiting bacterial growth.

A further major driver of antimicrobial resistance is horizontal gene transfer (HGT), which enables the rapid acquisition and dissemination of resistance genes across bacterial species. Through conjugation, transformation, and transduction, bacteria integrate foreign genetic material, enhancing their ability to withstand antibiotic treatments. Gauba et al. [44] highlight the prevalence of plasmid-mediated resistance in Gram-negative pathogens, particularly through integrons and transposons, which accelerate bacterial adaptation. Furthermore, Miller and Arias [45] emphasize the critical role of HGT in the evolution of ESKAPE pathogens (*Enterococcus faecium*, *Staphylococcus aureus*, *Klebsiella pneumoniae*, *Acinetobacter baumannii*, *Pseudomonas aeruginosa*, and *Enterobacter* spp.), which account for a substantial proportion of healthcare-associated infections worldwide.

The combination of these resistance strategies—including enzymatic degradation, efflux pump activation, biofilm formation, target modification, and horizontal gene transfer—underscores the extraordinary ability of bacterial pathogens to adapt under antimicrobial pressure [46]. This highlights the urgent need for innovative therapeutic interventions, such as next-generation β-lactamase inhibitors, efflux pump blockers, antibiofilm agents, and CRISPR-based precision antimicrobials. Insights from Rajput et al. [40], Gaurav et al. [41], Sharma et al. [42], Brdová et al. [43], and Gauba and Rahman [44] provide a strong foundation for the development of novel antimicrobial strategies to combat this escalating global threat.

A comprehensive overview of the primary bacterial resistance mechanisms is presented in Table 2, categorizing them by their molecular and genetic adaptations. These mechanisms include enzymatic inactivation, efflux pump activity, target modification, reduced membrane permeability, biofilm formation, and lipid modification. Each resistance strategy is associated with specific antibiotic classes and plays a critical role in bacterial survival against antimicrobial therapies. A deep understanding of these resistance mechanisms is essential for guiding the development of next-generation antibiotics, optimizing antimicrobial stewardship strategies, and effectively curbing the global spread of multidrug-resistant pathogens.

## 7. Innovative Approaches to Antibacterial Therapy

### 7.1. Bacteriophage Therapy

**Figure 2:**
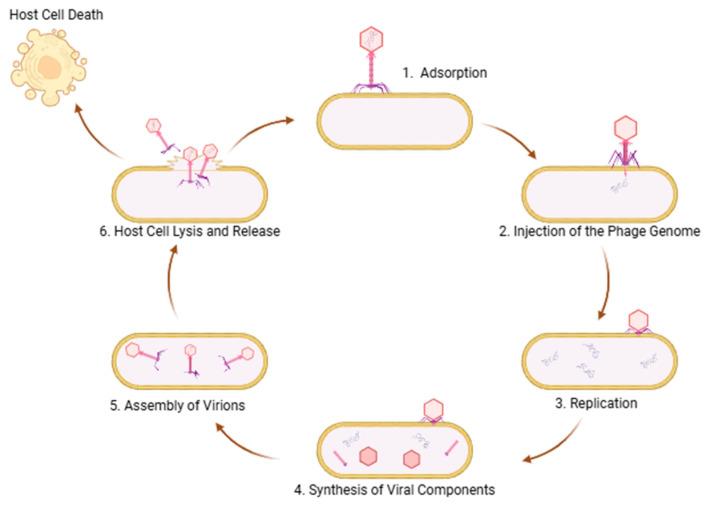
Schematic representation of the lytic cycle of a bacteriophage in the context of phage therapy. (1) The phage attaches to specific receptors on the bacterial surface (adsorption). (2) It injects its genome into the bacterium (injection). (3) The viral genetic material is replicated using the host cell’s machinery (genome replication). (4) Viral structural proteins and other components are synthesized (component synthesis). (5) The newly formed genomes and proteins assemble into complete virions (virion assembly). (6) Finally, the bacterial cell is lysed, releasing numerous new phages capable of infecting other bacteria (lysis and release).

Bacteriophages, or phages, are viruses that specifically infect and lyse bacteria. Bacteriophage therapy represents a highly targeted approach to combating bacterial infections, offering a promising alternative to conventional antibiotics in the face of the growing antimicrobial resistance (AMR) crisis [63,64,65,66,67]. Unlike broad-spectrum antibiotics, bacteriophages specifically infect and lyse bacterial strains without disrupting beneficial microbiota, thereby preserving the ecological balance and minimizing secondary infections. This level of specificity aligns with the principles of precision medicine, helping to maintain microbiome diversity while effectively eliminating pathogenic bacteria [68,69]. Phages are particularly advantageous in treating biofilm-associated infections, which exhibit significant resistance to both antibiotics and host immune responses. By producing extracellular polymer-degrading enzymes, phages can penetrate biofilms and lyse the bacteria within, making them especially valuable for managing infections related to medical devices, chronic wounds, and conditions such as cystic fibrosis [70,71]. Figure 2 provides a schematic overview of the bacteriophage lytic cycle. Their evolutionary adaptability further strengthens their therapeutic potential, as they co-evolve with bacterial hosts, sustaining their efficacy even in the face of bacterial resistance. This continuous evolutionary arms race offers long-term prospects for their clinical application while informing the refinement of phage therapies through ongoing research on bacterium–phage interactions [72]. Despite their potential, regulatory challenges have hindered widespread adoption. The highly personalized nature of phage therapy, which often requires strain-specific phages, presents logistical and cost-related challenges. Additionally, regulatory frameworks are still evolving to address standardization, safety, and efficacy concerns. Developing extensive phage libraries and rapid diagnostic tools is essential for making phage therapy more scalable and clinically feasible [73,74,75,76,77,78]. With continued advancements in research and technology, phage therapy holds the potential to become a transformative approach in the fight against AMR and in the broader field of microbiome-targeted therapies.

### 7.2. Antimicrobial Peptides (AMPs)

Antimicrobial peptides (AMPs) are another promising alternative to conventional antibiotics due to their ability to disrupt bacterial membranes and their broad-spectrum antimicrobial activity [79]. These naturally occurring molecules, found in various organisms, including amphibians [80,81], exhibit rapid bactericidal action by destabilizing bacterial membranes and interfering with essential cellular functions [82]. As described by Yang et al. [80], their mechanism extends beyond membrane disruption to include targeting bacterial proteins, making them highly versatile therapeutic agents. Efforts to enhance the therapeutic potential of AMPs have led to the development of synthetic AMPs, which address the stability issues of naturally occurring peptides. Xu et al. [83] detail methods such as chemical modifications and the incorporation of unnatural amino acids to improve pharmacokinetics, enhance structural stability under physiological conditions, and minimize cytotoxicity, thereby broadening their clinical applicability. Another innovation involves hybrid strategies that combine AMPs with conventional antibiotics. Bellucci et al. [84] demonstrate that AMP-antibiotic combinations exhibit synergistic effects, where AMPs disrupt bacterial membranes, enhancing antibiotic penetration and overall antimicrobial efficacy [82]. This approach is particularly valuable in overcoming bacterial resistance mechanisms. However, high production costs and enzymatic degradation remain significant challenges to widespread AMP adoption. Innovations in synthesis and delivery mechanisms, such as nanoparticle encapsulation and the development of peptidomimetics, are actively being explored to improve bioavailability and protect AMPs from degradation [83,84]. Advances in synthetic biology, chemical engineering, and hybrid therapies continue to refine AMPs, positioning them as next-generation antimicrobial agents with exceptional efficacy and safety profiles.

### 7.3. Nanotechnology-Based Solutions

Nanotechnology offers revolutionary approaches to combat multidrug-resistant (MDR) bacteria by exploiting the unique properties of nanoscale materials. Metallic nanoparticles, such as silver and gold, exhibit potent antibacterial effects, as they can disrupt bacterial membranes, compromise structural integrity, and generate reactive oxygen species (ROS), effectively inhibiting bacterial growth, particularly in MDR strains [85,86,87,88,89]. Nanotechnology has also transformed drug delivery systems by enhancing antibiotic stability, bioavailability, and precision. Nanocarriers enable antibiotics such as gentamicin and vancomycin to be delivered directly to infection sites, reducing systemic side effects and slowing resistance development, as demonstrated in studies by Pisani et al. [90]. Another vital application is the development of antibacterial coatings for medical devices, which are instrumental in preventing bacterial colonization and biofilm formation—major causes of device-associated infections. Coatings incorporating low-fouling polymers and selenium nanoparticles provide sustained antibacterial activity while ensuring biocompatibility, significantly reducing infection risks in surgical implants and other medical devices [90,91,92]. Despite their potential, concerns regarding the cytotoxicity and environmental impact of nanoparticles remain key challenges. To address these issues, rigorous safety assessments and the development of biodegradable or environmentally friendly nanomaterials are essential for ensuring long-term sustainability and clinical applicability [93,94]. By overcoming these limitations, nanotechnology continues to pave the way for safer and more effective solutions to combat MDR bacterial infections.

### 7.4. Microbial Extracellular Vesicles (EVs)

Microbial extracellular vesicles (EVs) have emerged as critical players in microbial communication, resistance gene dissemination, and biofilm development [95]. These nanoscale lipid bilayer structures carry diverse biomolecules, including nucleic acids, proteins, and lipids, facilitating interactions between microbial communities and host cells [96]. Their role in modulating host–pathogen interactions, shaping infection dynamics, and contributing to antibiotic resistance makes them a promising therapeutic target [97]. EVs enable bacteria to coordinate behaviors such as virulence factor production, stress responses, and biofilm formation, all of which enhance bacterial survival under antibiotic pressure [98]. Studies have revealed their role in interspecies and inter-kingdom communication, significantly influencing host immune responses and microbial ecology [97,98,99,100]. One of the major concerns surrounding EVs is their role in horizontal gene transfer (HGT), allowing bacteria to efficiently spread antibiotic resistance genes. Johnston et al. [101] demonstrated that *Pseudomonas aeruginosa* EVs can transfer plasmid DNA, accelerating the spread of multidrug resistance within microbial communities. Additionally, EVs play a crucial role in biofilm dynamics, reinforcing biofilm integrity while promoting the exchange of resistance determinants, making biofilm-associated infections particularly challenging to treat [102]. Targeting EVs offers novel therapeutic opportunities, including inhibiting EV biogenesis or degrading vesicle components to disrupt resistance gene dissemination and biofilm stability, thereby enhancing antibiotic efficacy [101,103,104]. Advances in related fields, such as cancer therapy, have demonstrated the feasibility of using EVs for targeted drug and genetic material delivery, inspiring potential applications in microbial systems [105,106,107]. Despite their promise, challenges remain regarding large-scale EV production, specificity in therapeutic applications, and mitigating unintended immune responses [108]. Developing robust methodologies for EV isolation, engineering, and targeted intervention, alongside thorough safety studies, is critical for translating EV research into viable antimicrobial therapies [109]. Multidisciplinary collaboration between researchers, clinicians, and policymakers is necessary to navigate ethical and regulatory considerations and fully harness the potential of EV-based interventions in combatting persistent infections and AMR [110,111,112].

### 7.5. CRISPR-Based Antimicrobials

CRISPR’s key advantage is its microbiome-sparing capability, ensuring that only pathogenic bacteria are targeted while commensal microbial populations remain unaffected. This precision is particularly valuable for preserving gut health and preventing secondary infections associated with dysbiosis [113,114,115,116]. The adaptability of CRISPR extends to diverse bacterial strains and clinical contexts, allowing for the fine-tuning of therapeutic applications. Vialetto et al. [117] emphasize the need for optimizing factors such as nuclease selection and guide RNA design to enhance specificity against pathogens like *Klebsiella pneumoniae*, demonstrating its versatility. CRISPR is also being explored as a tool for microbial engineering, not only to eliminate harmful traits but also to introduce beneficial genetic modifications, expanding its role beyond antimicrobial applications into biotechnology and therapeutic development [115]. The transformative potential of CRISPR-based antimicrobials lies in their ability to overcome the limitations of traditional antibiotics while providing scalable, targeted, and sustainable solutions to AMR. These technologies represent a paradigm shift in bacterial genome manipulation, offering molecular precision that redefines infection control and antimicrobial therapy [116]. However, achieving their full clinical potential requires further research to refine targeting mechanisms, ensure safety, and address ethical and ecological concerns [118,119]. Continued innovation and interdisciplinary collaboration will be key in harnessing CRISPR’s potential to combat bacterial infections and resistance effectively.

## 8. Future Directions

The future of antibacterial therapy depends on a multidisciplinary approach that integrates nanotechnology, combination therapies, personalized medicine, and global stewardship initiatives to combat antimicrobial resistance (AMR) [120]. Among the most promising emerging strategies, antimicrobial photodynamic therapy (aPDT) has garnered significant attention for its potential to tackle multidrug-resistant (MDR) bacterial infections without inducing resistance [121]. This technique utilizes photosensitizers that, when activated by light, generate reactive oxygen species (ROS) capable of inactivating bacteria, including those embedded in biofilms [91]. Recent studies have demonstrated the effectiveness of photosensitizing nanoassemblies, such as sulfobutylether-β-cyclodextrin-based systems, in enhancing the stability and efficacy of photosensitizers while simultaneously disrupting bacterial biofilms [91]. Furthermore, the development of Type I photodynamic therapy (PDT) has addressed a major limitation of conventional Type II PDT by eliminating its reliance on oxygen [122]. Type I PDT generates various ROS even in hypoxic environments, making it particularly effective against biofilms and deep-seated infections [123].

Recent advancements in laser-based antimicrobial strategies have also shown promise. A study investigating the viability and growth kinetics of vancomycin-resistant *Enterococcus faecalis* V583 (*E. faecalis* V583) following femtosecond laser irradiation (420–465 nm) demonstrated that optimized laser parameters can achieve a significant bactericidal effect without the need for exogenous photosensitizers [124]. The study identified 430 nm and 435 nm as the most effective wavelengths, achieving a ~2 log reduction (~98.6% and 98.3% inhibition, respectively) in viable bacterial counts at a fluence of 1000 J/cm^2^. Additionally, bacterial growth kinetics were progressively reduced with increasing energy density at 445 nm, with the most effective fluence being 1250 J/cm^2^. However, higher fluences (e.g., 2000 J/cm^2^) resulted in reduced efficacy due to the photobleaching of endogenous flavins. These findings highlight the importance of optimizing laser exposure parameters to maximize antimicrobial efficacy while minimizing unintended effects [124].

Beyond nanotechnology, combination therapies that integrate bacteriophages, antimicrobial peptides, CRISPR-based gene-editing tools, and aPDT have emerged as viable alternatives to conventional antibiotics. Hybrid multicomponent systems that combine PDT with antibiotics or nanoparticles have demonstrated synergistic effects, enhancing bacterial eradication while reducing the required antibiotic doses [120]. Among these, light-assisted nanotechnology-based combinatorial therapy is particularly promising, as it integrates PDT, nanoparticle drug delivery, and antibiotic therapy to improve bacterial clearance [125]. This approach has been shown to enhance the efficacy of individual antimicrobial agents while minimizing bacterial resistance, toxicity, and side effects [120]. Metallic and polymeric nanoparticles have been specifically designed to improve the delivery of antimicrobial agents, either by acting as carriers or by exerting intrinsic antibacterial activity [126]. Nanoparticles composed of gold (Au), silver (Ag), titanium dioxide (TiO₂), zinc oxide (ZnO), and copper oxide (CuO) have been widely studied for their ability to enhance the antibacterial effects of PDT by generating ROS and disrupting bacterial cell membranes [91].

Additionally, polymeric nanocarriers such as liposomes, dendrimers, and nanogels have been developed to enable controlled drug release and targeted bacterial eradication, particularly for biofilm-associated infections [120]. One of the most promising applications of nanotechnology-based light combination therapy is its ability to eradicate biofilms, which remain a significant challenge in treating MDR bacterial infections [127]. Biofilm-embedded bacteria exhibit up to 1000 times higher resistance to antibiotics than planktonic bacteria, and conventional treatments often fail due to limited drug penetration. However, light-activated nanomaterials can efficiently disrupt biofilms, allowing antibiotics or antimicrobial peptides to penetrate and eliminate pathogens [91]. Furthermore, engineered nano-antibiotics, in which antibiotics are conjugated with nanoparticles, have demonstrated significantly enhanced bacterial killing rates compared to free antibiotics [128]. These systems improve drug bioavailability, reduce toxicity, and prevent bacterial resistance by ensuring that antibiotics are delivered directly to infection sites, thereby optimizing therapeutic efficacy [120].

Advancements in personalized medicine and artificial intelligence-driven diagnostics are also transforming AMR management by enabling precise and tailored therapeutic interventions [129]. Machine learning-based predictive modeling has enhanced treatment efficacy by identifying optimal drug combinations, including PDT applications, based on bacterial genetic profiles and patient-specific factors [91]. Addressing AMR also necessitates a global commitment to sustainable antimicrobial use and environmental stewardship. Integrating aPDT into antimicrobial stewardship programs has the potential to reduce reliance on conventional antibiotics and limit the selective pressure that drives resistance evolution [120]. Additionally, the use of engineered microbial consortia and the enzymatic degradation of antibiotics in wastewater, combined with light-based disinfection strategies, could minimize environmental contamination and mitigate the spread of resistance genes [91].

The convergence of nanotechnology, combination therapies, antimicrobial photodynamic therapy, personalized medicine, and global antimicrobial stewardship represents a transformative shift in the fight against AMR [130]. These innovations have the potential to redefine infection management by integrating interdisciplinary research, optimizing regulatory frameworks, and fostering investment in next-generation antimicrobial technologies. Strengthening international collaborations and fostering public–private partnerships will be crucial in accelerating research and ensuring equitable access to novel antimicrobial solutions, particularly in low- and middle-income countries, where the burden of AMR is disproportionately high [91]. By advancing these strategies, the global community can not only curb the spread of resistance but also safeguard the long-term efficacy of antimicrobial therapies for future generations [120].

## 9. Conclusions

The escalating crisis of antimicrobial resistance (AMR) demands transformative solutions, with emerging innovations presenting unprecedented opportunities to combat this global threat [4]. Groundbreaking advancements in infection management, including bacteriophage therapy, nanotechnology-based interventions, peptide dendrimer-based agents, and CRISPR-based antimicrobials, are redefining the landscape of antibacterial treatment [4]. Bacteriophage therapy, as highlighted by Strathdee et al. [131], employs bacteriophages to selectively target and eliminate bacterial pathogens, effectively bypassing conventional resistance mechanisms. Advances in genetic engineering have further refined phage specificity and safety, enabling their integration into clinical applications. Complementing this, nanotechnology-based solutions, as explored by Solanki et al. [132] and Ioannou et al. [133], facilitate precise drug delivery through nanoparticles and dendrimer-based carriers. These approaches enhance bioavailability, minimize toxicity, and address the limitations of traditional antibiotics, particularly in treating biofilm-associated infections, which often exhibit heightened resistance [4].

Peptide dendrimer-based agents, investigated by Paul et al. [134], provide a highly adaptable platform for disrupting bacterial membranes and inhibiting biofilm formation. These versatile molecules offer targeted, efficient, and sustainable antibacterial strategies, marking a paradigm shift in AMR management [135]. However, the successful implementation of these novel therapies depends on the establishment of robust global frameworks and interdisciplinary collaboration. Overcoming economic, logistical, and regulatory obstacles is crucial, necessitating international coordination, enhanced surveillance systems, increased research funding, and stronger public–private partnerships [136]. Furthermore, reinforcing antibiotic stewardship remains vital to preserving the efficacy of both existing and emerging therapies, ensuring their responsible and sustainable use [4].

Looking to the future, the integration of artificial intelligence (AI)-driven diagnostics, predictive modeling, and personalized medicine promises to further revolutionize AMR management. AI applications can refine treatment strategies by identifying optimal antimicrobial combinations tailored to both patient-specific and pathogen-specific factors, significantly enhancing therapeutic precision [4]. In addition, innovations such as light-assisted nanotechnology, microbial extracellular vesicles, and CRISPR-based gene editing hold immense potential for disrupting bacterial resistance mechanisms while simultaneously preserving the integrity of the host microbiome [4]. These approaches not only expand the arsenal of available antimicrobial therapies but also open new avenues for developing highly specific and minimally disruptive treatments.

A proactive global commitment to innovation and collaboration, backed by sustained investment, presents a viable strategy for controlling the AMR crisis while simultaneously reshaping modern healthcare. This vision not only addresses the immediate challenges of resistance but also fosters a sustainable and resilient healthcare system, ensuring the long-term efficacy of antimicrobial therapies for future generations [136]. By embracing these emerging technologies and reinforcing coordinated global efforts, the international community can establish a more effective and adaptable framework to combat AMR and secure the future of infectious disease management.

## Figures and Tables

**Table 1:** PRISMA 2020 compliance checklist [10]: a summary of key reporting items, their descriptions, and the extent of compliance in this study. This checklist ensures methodological rigor and completeness in the systematic review process.

Section	Requirement	Present in the Manuscript
Title	Identify as a systematic review, meta-analysis, or both.	Yes
Abstract	Structured summary including background, methods, results, and conclusion.	Yes
Rationale	Describe the rationale for the review.	Yes
Objectives	Provide an explicit statement of the objectives.	No
Eligibility Criteria	Specify study eligibility criteria.	No
Information Sources	Specify all information sources (e.g., databases and dates).	Yes
Search Strategy	Present a full search strategy for at least one database.	No
Selection Process	Describe the selection process (screening, eligibility, and inclusion).	No
Data Collection Process	Describe methods of data extraction and management.	No
Data Items	List all variables and outcomes collected.	No
Study Risk of Bias Assessment	Describe methods for assessing risk of bias.	No
Effect Measures	Describe all methods for effect estimation.	No
Synthesis Methods	Specify how the results were synthesized.	No
Certainty Assessment	Assess certainty in evidence (e.g., GRADE framework).	No
Results	Report the number of included studies and characteristics.	Yes
Discussion	Discuss results in the context of limitations and strengths.	No
Funding	Disclose funding sources and conflicts of interest.	Yes

**Table 2:** Key mechanisms of bacterial resistance to various antibiotic classes.

Mechanism of Resistance	Targeted Antibiotic Classes	Details and Examples	References
Alteration of Target Proteins	Beta-lactams (Penicillins, Cephalosporins, Carbapenems, and Monobactams); Glycopeptides (Vancomycin and Teicoplanin)	Methicillin-resistant *Staphylococcus aureus* (MRSA) develops resistance through mecA gene expression, encoding an alternative penicillin-binding protein (PBP2a) with reduced affinity for β-lactam antibiotics, rendering them ineffective. Similarly, vancomycin resistance in *Enterococcus faecium* and *Enterococcus faecalis* is mediated by van gene clusters, leading to the substitution of the terminal D-Ala-D-Ala in peptidoglycan precursors with D-Ala-D-Lac, which significantly reduces the binding efficacy of vancomycin, thereby conferring high-level resistance.	[45,46,47]
Enzymatic Inactivation	Aminoglycosides (Amikacin, Gentamicin, and Kanamycin); Beta-lactams (Penicillins and Cephalosporins)	β-lactamase production is a widespread resistance mechanism, particularly in Gram-negative bacteria. Extended-spectrum β-lactamases (ESBLs) such as TEM, SHV, and CTX-M hydrolyze oxyimino-cephalosporins, compromising the efficacy of cefotaxime, ceftazidime, and ceftriaxone. Additionally, carbapenem-resistant Enterobacterales (CRE) produce carbapenemases like KPC (*Klebsiella pneumoniae* carbapenemase), NDM (New Delhi metallo-β-lactamase), and OXA-48, which degrade even last-resort carbapenems, leading to treatment failures and limited therapeutic options.	[48,49,50]
Efflux Pump Activity	Macrolides (Erythromycin, Clarithromycin, and Azithromycin); Fluoroquinolones (Ciprofloxacin and Levofloxacin)	Multidrug efflux pumps significantly contribute to antibiotic resistance by reducing intracellular drug concentrations. The AcrAB-TolC efflux system in *Escherichia coli* and the MexAB-OprM system in *Pseudomonas aeruginosa* expel a wide range of antibiotics, including fluoroquinolones, macrolides, tetracyclines, and β-lactams. Additionally, the NorA efflux pump in *Staphylococcus aureus* is responsible for resistance to fluoroquinolones, whereas the Tet efflux family, including Tet(A) and Tet(K), confers resistance to tetracyclines by actively transporting the antibiotic out of the bacterial cell.	[51,52]
Reduced Permeability	Beta-lactams and Aminoglycosides	Gram-negative bacteria can restrict antibiotic entry by modifying outer membrane porins. The loss or downregulation of porins such as OmpF and OmpC in Enterobacteriaceae reduces susceptibility to β-lactams, particularly carbapenems, leading to resistance in *Klebsiella pneumoniae*, *Escherichia coli*, and *Acinetobacter baumannii*. Mutations in porin-coding genes further enhance resistance by decreasing drug influx, often in conjunction with efflux pump overexpression, which exacerbates antibiotic treatment failure.	[53,54]
Target Bypass	Folate Inhibitors (Trimethoprim and Sulfonamides)	Bacteria can develop resistance to folate synthesis inhibitors, such as trimethoprim and sulfonamides, by overproducing dihydrofolate reductase (DHFR) or acquiring mutations in the dhfr gene. The overexpression of an insensitive DHFR enzyme allows the bacterial cell to maintain folate biosynthesis even in the presence of the drug. Additionally, plasmid-encoded resistant DHFR variants, such as DHFR type I and type II in *Escherichia coli* and *Staphylococcus aureus*, display reduced affinity for trimethoprim while retaining enzymatic activity, effectively bypassing the inhibitory mechanism of these antimicrobial agents. Some Gram-negative pathogens, including *Klebsiella pneumoniae*, also acquire horizontally transferred resistance genes, such as dfrA, further exacerbating resistance to trimethoprim–sulfamethoxazole treatment.	[55,56]
Biofilm Formation	Broad-spectrum Antibiotics	Bacterial biofilms are highly structured, multicellular communities encased in a self-produced extracellular polymeric substance (EPS) matrix, which protects them from antimicrobial agents. The biofilm matrix, composed of polysaccharides, proteins, and extracellular DNA, physically restricts antibiotic penetration, leading to sublethal drug concentrations within deeper layers. Additionally, bacteria within biofilms exhibit an altered metabolic state, including reduced growth rates and the activation of stress response pathways, which enhance tolerance to antibiotics. *Pseudomonas aeruginosa*, a major biofilm-forming pathogen in chronic infections, employs quorum sensing (QS) to regulate biofilm development and increase resistance to β-lactams, aminoglycosides, and fluoroquinolones. Similarly, *Staphylococcus epidermidis* biofilms contribute to persistent infections in medical implants by increasing tolerance to vancomycin and rifampin, necessitating alternative therapeutic approaches.	[57,58]
Modification of Ribosomal Targets	Tetracyclines (Tetracycline and Doxycycline); Oxazolidinones (Linezolid)	Bacteria evade ribosome-targeting antibiotics by either modifying ribosomal RNA (rRNA) or employing ribosomal protection proteins (RPPs) to prevent antibiotic binding. In tetracycline-resistant bacteria, RPPs such as Tet(O) and Tet(M) dislodge tetracyclines from the 30S ribosomal subunit, restoring normal translation activity. Additionally, mutations in the 23S rRNA gene confer resistance to oxazolidinones like linezolid. In *Staphylococcus aureus* and *Enterococcus faecium*, mutations in domain V of 23S rRNA (G2576T) disrupt linezolid binding, reducing its inhibitory effect. The acquisition of cfr (chloramphenicol–florfenicol resistance) methyltransferase genes further modifies 23S rRNA, leading to resistance not only to linezolid but also to phenicols, streptogramins, and lincosamides, posing a significant challenge in clinical settings.	[59,60]
Lipid Modification	Cationic Peptides (Colistin and Polymyxin E)	The modification of lipopolysaccharides (LPS) in Gram-negative bacteria reduces the binding affinity of cationic antimicrobial peptides (CAMPs), such as colistin and polymyxin B. *Acinetobacter baumannii* and *Klebsiella pneumoniae* achieve this resistance through the addition of cationic groups (phosphoethanolamine or 4-amino-4-deoxy-L-arabinose) to lipid A, the lipid anchor of LPS, via the pmrAB and arn operon regulatory systems. These modifications neutralize the negative charge of LPS, diminishing electrostatic interactions with CAMPs and thereby reducing their antimicrobial activity. Additionally, mutations in the lpxC, lpxD, and lpxM genes can lead to the complete loss of LPS biosynthesis, conferring high-level colistin resistance in certain A. baumannii strains. This resistance mechanism is particularly concerning as it limits the efficacy of last-resort polymyxins in treating carbapenem-resistant infections.	[61,62]

## Data Availability

No new data were created or analyzed in this study.

## References

[1] Ferraz M.P. (2024). Antimicrobial Resistance: The Impact from and on Society According to One Health Approach. Societies.

[2] Ahmed S.K., Hussein S., Qurbani K., Ibrahim R.H., Fareeq A., Mahmood K.A., Mohamed M.G. (2024). Antimicrobial Resistance: Impacts, Challenges, and Future Prospects. J. Med. Surg. Public Health.

[3] Oliveira M., Antunes W., Mota S., Madureira-Carvalho Á., Dinis-Oliveira R.J., Dias da Silva D. (2024). An Overview of the Recent Advances in Antimicrobial Resistance. Microorganisms.

[4] Bergkessel M., Forte B., Gilbert I.H. (2023). Small-Molecule Antibiotic Drug Development: Need and Challenges. ACS Infect. Dis..

[5] Ho C.S., Wong C.T.H., Aung T.T., Lakshminarayanan R., Mehta J.S., Rauz S., McNally A., Kintses B., Peacock S.J., de la Fuente-Nunez C. (2025). Antimicrobial Resistance: A Concise Update. Lancet Microbe.

[6] Muteeb G., Rehman M.T., Shahwan M., Aatif M. (2023). Origin of Antibiotics and Antibiotic Resistance, and Their Impacts on Drug Development: A Narrative Review. Pharmaceuticals.

[7] Murugaiyan J., Kumar P.A., Rao G.S., Iskandar K., Hawser S., Hays J.P., Mohsen Y., Adukkadukkam S., Awuah W.A., Jose R.A. (2022). Progress in Alternative Strategies to Combat Antimicrobial Resistance: Focus on Antibiotics. Antibiotics.

[8] Chinemerem Nwobodo D., Ugwu M.C., Oliseloke Anie C., Al-Ouqaili M.T.S., Chinedu Ikem J., Victor Chigozie U., Saki M. (2022). Antibiotic Resistance: The Challenges and Some Emerging Strategies for Tackling a Global Menace. J. Clin. Lab. Anal..

[9] Kuchay R.A.H. (2024). Novel and Emerging Therapeutics for Antimicrobial Resistance: A Brief Review. Drug Discov. Ther..

[10] Page M.J., McKenzie J.E., Bossuyt P.M., Boutron I., Hoffmann T.C., Mulrow C.D., Shamseer L., Tetzlaff J.M., Akl E.A., Brennan S.E. (2021). The PRISMA 2020 Statement: An Updated Guideline for Reporting Systematic Reviews. BMJ.

[11] Coque T., Canton R., Pérez-Cobas A., Fernández-de-Bobadilla M., Baquero F. (2023). Antimicrobial Resistance in the Global Health Network: Known Unknowns and Challenges for Efficient Responses in the 21st Century. Microorganisms.

[12] Salam M.A., Al-Amin M.Y., Salam M.T., Pawar J.S., Akhter N., Rabaan A.A., Alqumber M.A.A. (2023). Antimicrobial Resistance: A Growing Serious Threat for Global Public Health. Healthcare.

[13] Miller S.A., Ferreira J.P., LeJeune J.T. (2022). Antimicrobial Use and Resistance in Plant Agriculture: A One Health Perspective. Agriculture.

[14] Kasimanickam V., Kasimanickam M., Kasimanickam R. (2021). Antibiotics Use in Food Animal Production: Escalation of Antimicrobial Resistance: Where Are We Now in Combating AMR?. Med. Sci..

[15] Okaiyeto S., Xiao H.-W., Mujumdar A., Sutar P., Ni J. (2024). Antibiotic Resistant Bacteria in Food Systems: Current Status, Resistance Mechanisms, and Mitigation Strategies. Agric. Commun..

[16] Endale H., Mathewos M., Abdeta D. (2023). Potential Causes of Spread of Antimicrobial Resistance and Preventive Measures in One Health Perspective-A Review. Infect. Drug Resist..

[17] Rodriguez J. (2024). One Health Ethics and the Ethics of Zoonoses: A Silent Call for Global Action. Vet. Sci..

[18] Koutsoumanis K., Allende A., Álvarez-Ordóñez A., Bolton D., Bover-Cid S., Chemaly M., Davies R., De Cesare A., Herman L., EFSA Panel on Biological Hazards (BIOHAZ) (2021). Role Played by the Environment in the Emergence and Spread of Antimicrobial Resistance (AMR) through the Food Chain. EFSA J..

[19] Majumder M.A., Rahman S., Cohall D., Bharatha A., Singh K., Haque M., Hilaire M. (2020). Antimicrobial Stewardship: Fighting Antimicrobial Resistance and Protecting Global Public Health. Infect. Drug Resist..

[20] Manyi-Loh C., Mamphweli S., Meyer E., Okoh A. (2018). Antibiotic Use in Agriculture and Its Consequential Resistance in Environmental Sources: Potential Public Health Implications. Molecules.

[21] Abdallah E.M., Alhatlani B.Y., de Paula Menezes R., Martins C.H. (2023). Back to Nature: Medicinal Plants as Promising Sources for Antibacterial Drugs in the Post-Antibiotic Era. Plants.

[22] Edelstein M., Lee L., Herten-Crabb A., Heymann D., Harper D. (2018). Strengthening Global Public Health Surveillance through Data and Benefit Sharing. Emerg. Infect. Dis..

[23] Ghimpețeanu O.M., Pogurschi E.N., Popa D.C., Dragomir N., Drăgotoiu T., Mihai O.D., Petcu C.D. (2022). Antibiotic Use in Livestock and Residues in Food—A Public Health Threat: A Review. Foods.

[24] Fernandez Miyakawa M., Casanova N., Kogut M. (2023). How Did Antibiotic Growth Promoters Increase Growth and Feed Efficiency in Poultry?. Poult. Sci..

[25] Caneschi A., Bardhi A., Barbarossa A., Zaghini A. (2023). The Use of Antibiotics and Antimicrobial Resistance in Veterinary Medicine, a Complex Phenomenon: A Narrative Review. Antibiotics.

[26] Pandey S., Doo H., Keum G., Kim E.S., Kwak J., Ryu S., Choi Y., Kang J., Kim S., Lee N. (2023). Antibiotic Resistance in Livestock, Environment and Humans: One Health Perspective. J. Anim. Sci. Technol..

[27] Sun Z., Hong W., Xue C., Dong N. (2024). A Comprehensive Review of Antibiotic Resistance Gene Contamination in Agriculture: Challenges and AI-Driven Solutions. Sci. Total Environ..

[28] Mann A., Nehra K., Rana J.S., Twinkle T. (2021). Antibiotic Resistance in Agriculture: Perspectives on Upcoming Strategies to Overcome Upsurge in Resistance. Curr. Res. Microb. Sci..

[29] Xu C., Aqib A.I., Fatima M., Muneer S., Zaheer T., Peng S., Ibrahim E.H., Li K. (2024). Deciphering the Potential of Probiotics in Vaccines. Vaccines.

[30] Kazemifard N., Dehkohneh A., Baradaran Ghavami S. (2022). Probiotics and Probiotic-Based Vaccines: A Novel Approach for Improving Vaccine Efficacy. Front. Med..

[31] Dutescu I.A., Hillier S.A. (2021). Encouraging the Development of New Antibiotics: Are Financial Incentives the Right Way Forward? A Systematic Review and Case Study. Infect. Drug Resist..

[32] Westgeest A.C., Schippers E.F., Sijbom M., Visser L.G., de Boer M.G.J., Numans M.E., Lambregts M.M.C., on behalf of the MRSA Network Holland West (2022). Exploring the Barriers in the Uptake of the Dutch MRSA ‘Search and Destroy’ Policy Using the Cascade of Care Approach. Antibiotics.

[33] Anderson M., Panteli D., Kessel R., Ljungqvist G., Colombo F., Mossialos E. (2023). Challenges and Opportunities for Incentivising Antibiotic Research and Development in Europe. Lancet Reg. Health. Eur..

[34] Hutchings M.I., Truman A.W., Wilkinson B. (2019). Antibiotics: Past, Present and Future. Curr. Opin. Microbiol..

[35] Aslam B., Asghar R., Muzammil S., Shafique M., Siddique A., Khurshid M., Ijaz M., Rasool M., Chaudhry T., Aamir A. (2024). AMR and Sustainable Development Goals: At a Crossroads. Glob. Health.

[36] Sarpong D., Boakye D., Ofosu G., Botchie D. (2023). The Three Pointers of Research and Development (R&D) for Growth-Boosting Sustainable Innovation System. Technovation.

[37] Simpkin V.L., Renwick M.J., Kelly R., Mossialos E. (2017). Incentivising Innovation in Antibiotic Drug Discovery and Development: Progress, Challenges and next Steps. J. Antibiot..

[38] Belay W.Y., Getachew M., Tegegne B.A., Teffera Z.H., Dagne A., Zeleke T.K., Abebe R.B., Gedif A.A., Fenta A., Yirdaw G. (2024). Mechanism of Antibacterial Resistance, Strategies and next-Generation Antimicrobials to Contain Antimicrobial Resistance: A Review. Front. Pharmacol..

[39] Kruk M.E., Gage A.D., Arsenault C., Jordan K., Leslie H.H., Roder-DeWan S., Adeyi O., Barker P., Daelmans B., Doubova S.V. (2018). High-Quality Health Systems in the Sustainable Development Goals Era: Time for a Revolution. Lancet Glob. Health.

[40] Rajput P., Nahar K.S., Rahman K.M. (2024). Evaluation of Antibiotic Resistance Mechanisms in Gram-Positive Bacteria. Antibiotics.

[41] Gaurav A., Bakht P., Saini M., Pandey S., Pathania R. (2023). Role of Bacterial Efflux Pumps in Antibiotic Resistance, Virulence, and Strategies to Discover Novel Efflux Pump Inhibitors. Microbiology.

[42] Sharma S., Mohler J., Mahajan S.D., Schwartz S.A., Bruggemann L., Aalinkeel R. (2023). Microbial Biofilm: A Review on Formation, Infection, Antibiotic Resistance, Control Measures, and Innovative Treatment. Microorganisms.

[43] Brdová D., Ruml T., Viktorová J. (2024). Mechanism of Staphylococcal Resistance to Clinically Relevant Antibiotics. Drug Resist. Updates.

[44] Gauba A., Rahman K.M. (2023). Evaluation of Antibiotic Resistance Mechanisms in Gram-Negative Bacteria. Antibiotics.

[45] Miller W.R., Arias C.A. (2024). ESKAPE Pathogens: Antimicrobial Resistance, Epidemiology, Clinical Impact and Therapeutics. Nat. Rev. Microbiol..

[46] Khan R.T., Sharma V., Khan S.S., Rasool S. (2024). Prevention and Potential Remedies for Antibiotic Resistance: Current Research and Future Prospects. Front. Microbiol..

[47] Pozzi C. (2020). Editorial for the Special Issue: “Targeting β-Lactamases to Fight Bacterial Resistance to β-Lactam Antibiotics”. Antibiotics.

[48] Kapoor G., Saigal S., Elongavan A. (2017). Action and Resistance Mechanisms of Antibiotics: A Guide for Clinicians. J. Anaesthesiol. Clin. Pharmacol..

[49] Sawa T., Kooguchi K., Moriyama K. (2020). Molecular Diversity of Extended-Spectrum β-Lactamases and Carbapenemases, and Antimicrobial Resistance. J. Intensive Care.

[50] Lin W.-T., Lai C.-C., Cheong C.-U. (2019). Novel β-Lactam/β-Lactamase Combination Versus Meropenem for Treating Nosocomial Pneumonia. Antibiotics.

[51] Magaña A.J., Sklenicka J., Pinilla C., Giulianotti M., Chapagain P., Santos R., Ramirez M.S., Tolmasky M.E. (2023). Restoring Susceptibility to Aminoglycosides: Identifying Small Molecule Inhibitors of Enzymatic Inactivation. RSC Med. Chem..

[52] Zárate S., Claure M., Benito-Arenas R., Revuelta J., Santana A., Bastida A. (2018). Overcoming Aminoglycoside Enzymatic Resistance: Design of Novel Antibiotics and Inhibitors. Molecules.

[53] Bialvaei A.Z., Kafil H.S. (2015). Colistin, Mechanisms and Prevalence of Resistance. Curr. Med. Res. Opin..

[54] Aghapour Z., Gholizadeh P., Ganbarov K., Bialvaei A.Z., Mahmood S.S., Tanomand A., Yousefi M., Asgharzadeh M., Yousefi B., Kafil H.S. (2019). Molecular Mechanisms Related to Colistin Resistance in Enterobacteriaceae. Infect. Drug Resist..

[55] Byrd B., Zenick B., Rocha-Granados M., Englander H., Hare P., LaGree T., Power A., Mok W. (2021). The AcrAB-TolC Efflux Pump Impacts Persistence and Resistance Development in Stationary-Phase Escherichia Coli Following Delafloxacin Treatment. Antimicrob. Agents Chemother..

[56] Gogoi I., Puzari M., Chetia P. (2023). Porin-Mediated Carbapenem Resistance in Klebsiella Pneumoniae: An Alarming Threat to Global Health. Curr. Clin. Microbiol. Rep..

[57] Elías-López C., Muñoz-Rosa M., Guzmán-Puche J., Pérez-Nadales E., Chicano-Galvez E., Martínez-Martínez L. (2024). Porin Expression in Clinical Isolates of Klebsiella Pneumoniae: A Comparison of SDS-PAGE and MALDI-TOF/MS and Limitations of Whole Genome Sequencing Analysis. Ann. Clin. Microbiol. Antimicrob..

[58] Ambrose S., Hall R. (2021). dfrA Trimethoprim Resistance Genes Found in Gram-Negative Bacteria: Compilation and Unambiguous Numbering. J. Antimicrob. Chemother..

[59] Skold O. (2001). Resistance to Trimethoprim and Sulfonamides. Vet. Res..

[60] Azeem K., Fatima S., Ali A., Ubaid A., Husain F.M., Abid M. (2025). Biochemistry of Bacterial Biofilm: Insights into Antibiotic Resistance Mechanisms and Therapeutic Intervention. Life.

[61] Grooters K.E., Ku J.C., Richter D.M., Krinock M.J., Minor A., Li P., Kim A., Sawyer R., Li Y. (2024). Strategies for Combating Antibiotic Resistance in Bacterial Biofilms. Front. Cell. Infect. Microbiol..

[62] Li W., Atkinson G.C., Thakor N.S., Allas Ü., Lu C., Chan K.-Y., Tenson T., Schulten K., Wilson K.S., Hauryliuk V. (2013). Mechanism of Tetracycline Resistance by Ribosomal Protection Protein Tet(O). Nat. Commun..

[63] Han X., Zou G., Liu J., Yang C., Du X., Chen G., Sun Z., Zhang X., Sun Y., Zhang W. (2022). Mechanisms of Linezolid Resistance in Staphylococcus Capitis with the Novel Mutation C2128T in the 23S rRNA Gene in China. BMC Microbiology.

[64] Islam M.M., Jung D.E., Shin W.S., Oh M.H. (2024). Colistin Resistance Mechanism and Management Strategies of Colistin-Resistant Acinetobacter Baumannii Infections. Pathogens.

[65] Novović K., Jovčić B. (2023). Colistin Resistance in Acinetobacter Baumannii: Molecular Mechanisms and Epidemiology. Antibiotics.

[66] Fowoyo P.T. (2024). Phage Therapy: Clinical Applications, Efficacy, and Implementation Hurdles. Open Microbiol. J..

[67] Pirnay J.-P., Djebara S., Steurs G., Griselain J., Cochez C., De Soir S., Glonti T., Spiessens A., Vanden Berghe E., Green S. (2024). Personalized Bacteriophage Therapy Outcomes for 100 Consecutive Cases: A Multicentre, Multinational, Retrospective Observational Study. Nat. Microbiol..

[68] Singh S., Nath G., Maheshwari A. (2024). Bacteriophages. Newborn.

[69] Olawade D., Fapohunda O., Egbon E., Ebiesuwa O., Usman S., Faronbi A.O., Fidelis S. (2024). Phage Therapy: A Targeted Approach to Overcoming Antibiotic Resistance. Microb. Pathog..

[70] Meneses L., Brandão A., Coenye T., Braga A., Pires D., Azeredo J. (2023). A Systematic Review of the Use of Bacteriophages for in Vitro Biofilm Control. Eur. J. Clin. Microbiol. Infect. Dis..

[71] de la Cuesta-Zuluaga J., Boldt L., Maier L. (2024). Response, Resistance, and Recovery of Gut Bacteria to Human-Targeted Drug Exposure. Cell Host Microbe.

[72] Cao C., Yue S., Lu A., Liang C. (2024). Host-Gut Microbiota Metabolic Interactions and Their Role in Precision Diagnosis and Treatment of Gastrointestinal Cancers. Pharmacol. Res..

[73] Mayorga-Ramos A., Carrera-Pacheco S.E., Barba-Ostria C., Guamán L.P. (2024). Bacteriophage-Mediated Approaches for Biofilm Control. Front. Cell. Infect. Microbiol..

[74] Gordon M., Ramirez P. (2024). Efficacy and Experience of Bacteriophages in Biofilm-Related Infections. Antibiotics.

[75] Fortuna M., Barbour M., Zaman L., Hall A., Buckling A., Bascompte J. (2019). Coevolutionary Dynamics Shape the Structure of Bacteria-phage Infection Networks. Evolution.

[76] Cui L., Watanabe S., Miyanaga K., Kiga K., Sasahara T., Aiba Y., Tan X.-E., Veeranarayanan S., Thitiananpakorn K., Nguyen H.M. (2024). A Comprehensive Review on Phage Therapy and Phage-Based Drug Development. Antibiotics.

[77] Zalewska-Piątek B. (2023). Phage Therapy—Challenges, Opportunities and Future Prospects. Pharmaceuticals.

[78] Xu H., Cao B., Li Y., Mao C. (2020). Phage Nanofibers in Nanomedicine: Biopanning for Early Diagnosis, Targeted Therapy, and Proteomics Analysis. Wiley Interdiscip. Rev. Nanomed. Nanobiotechnol..

[79] Gagandeep K.R., Balenahalli Narasingappa R., Vishnu Vyas G. (2024). Unveiling Mechanisms of Antimicrobial Peptide: Actions beyond the Membranes Disruption. Heliyon.

[80] Yang J., Zhang J., Feng Z., Ma Y. (2025). The Role and Mechanisms of Antimicrobial Peptides in Overcoming Multidrug-Resistant Bacteria. Molecules.

[81] Canè C., Tammaro L., Duilio A., Di Somma A. (2024). Investigation of the Mechanism of Action of AMPs from Amphibians to Identify Bacterial Protein Targets for Therapeutic Applications. Antibiotics.

[82] Mihaylova-Garnizova R., Davidova S., Hodzhev Y., Satchanska G. (2024). Antimicrobial Peptides Derived from Bacteria: Classification, Sources, and Mechanism of Action against Multidrug-Resistant Bacteria. Int. J. Mol. Sci..

[83] Xu S., Tan P., Tang Q., Wang T., Ding Y., Fu H., Zhang Y., Zhou C., Song M., Tang Q. (2023). Enhancing the Stability of Antimicrobial Peptides: From Design Strategies to Applications. Chem. Eng. J..

[84] Bellucci M.C., Romani C., Sani M., Volonterio A. (2024). Dual Antibiotic Approach: Synthesis and Antibacterial Activity of Antibiotic–Antimicrobial Peptide Conjugates. Antibiotics.

[85] Taheri-Araghi S. (2024). Synergistic Action of Antimicrobial Peptides and Antibiotics: Current Understanding and Future Directions. Front. Microbiol..

[86] Min K.H., Kim K.H., Ki M.-R., Pack S.P. (2024). Antimicrobial Peptides and Their Biomedical Applications: A Review. Antibiotics.

[87] Wang R., Wang T., Zhuo L., Wei J., Fu X., Zou Q., Yao X. (2024). Diff-AMP: Tailored Designed Antimicrobial Peptide Framework with All-in-One Generation, Identification, Prediction and Optimization. Brief. Bioinform..

[88] Aguilar-Garay R., Lara-Ortiz L.F., Campos-López M., Gonzalez-Rodriguez D.E., Gamboa-Lugo M.M., Mendoza-Pérez J.A., Anzueto-Ríos Á., Nicolás-Álvarez D.E. (2024). A Comprehensive Review of Silver and Gold Nanoparticles as Effective Antibacterial Agents. Pharmaceuticals.

[89] Mammari N., Lamouroux E., Boudier A., Duval R. (2022). Current Knowledge on the Oxidative-Stress-Mediated Antimicrobial Properties of Metal-Based Nanoparticles. Microorganisms.

[90] Pisani S., Tufail S., Rosalia M., Dorati R., Genta I., Chiesa E., Conti B. (2024). Antibiotic-Loaded Nano-Sized Delivery Systems: An Insight into Gentamicin and Vancomycin. J. Funct. Biomater..

[91] Hetta H.F., Ramadan Y.N., Al-Harbi A.I., Ahmed E.A., Battah B., Abd Ellah N.H., Zanetti S., Donadu M.G. (2023). Nanotechnology as a Promising Approach to Combat Multidrug Resistant Bacteria: A Comprehensive Review and Future Perspectives. Biomedicines.

[92] Huang Y., Guo X., Wu Y., Chen X., Feng L., Xie N., Shen G. (2024). Nanotechnology’s Frontier in Combatting Infectious and Inflammatory Diseases: Prevention and Treatment. Signal Transduct. Target. Ther..

[93] Negut I., Albu C., Bita B. (2024). Advances in Antimicrobial Coatings for Preventing Infections of Head-Related Implantable Medical Devices. Coatings.

[94] Sahoo J., Sarkhel S., Mukherjee N., Jaiswal A. (2022). Nanomaterial-Based Antimicrobial Coating for Biomedical Implants: New Age Solution for Biofilm-Associated Infections. ACS Omega.

[95] Li F., Huang T., Pasic P., Easton C.D., Voelcker N.H., Heath D.E., O’Brien-Simpson N.M., O’Connor A.J., Thissen H. (2023). One Step Antimicrobial Coatings for Medical Device Applications Based on Low Fouling Polymers Containing Selenium Nanoparticles. Chem. Eng. J..

[96] Kumah E., Djou Fopa R., Harati S., Boadu P., Zohoori F., Pak T. (2023). Human and Environmental Impacts of Nanoparticles: A Scoping Review of the Current Literature. BMC Public Health.

[97] Kumari R., Suman K., Karmakar S., Mishra V., Lakra S.G., Saurav G.K., Mahto B.K. (2023). Regulation and Safety Measures for Nanotechnology-Based Agri-Products. Front. Genome Ed..

[98] Jiang B., Lai Y., Xiao W., Zhong T., Liu F., Gong J., Huang J. (2024). Microbial Extracellular Vesicles Contribute to Antimicrobial Resistance. PLOS Pathog..

[99] Jahromi L.P., Fuhrmann G. (2021). Bacterial Extracellular Vesicles: Understanding Biology Promotes Applications as Nanopharmaceuticals. Adv. Drug Deliv. Rev..

[100] Fang Y., Wang Z., Liu X., Tyler B.M. (2022). Biogenesis and Biological Functions of Extracellular Vesicles in Cellular and Organismal Communication with Microbes. Front. Microbiol..

[101] Johnston E.L., Zavan L., Bitto N.J., Petrovski S., Hill A.F., Kaparakis-Liaskos M. (2023). Planktonic and Biofilm-Derived Pseudomonas Aeruginosa Outer Membrane Vesicles Facilitate Horizontal Gene Transfer of Plasmid DNA. Microbiol. Spectr..

[102] Zaborowska M., Taulé Flores C., Vazirisani F., Shah F.A., Thomsen P., Trobos M. (2020). Extracellular Vesicles Influence the Growth and Adhesion of Staphylococcus Epidermidis Under Antimicrobial Selective Pressure. Front. Microbiol..

[103] Nahui Palomino R., Vanpouille C., Costantini P.E., Margolis L. (2021). Microbiota–Host Communications: Bacterial Extracellular Vesicles as a Common Language. PLoS Pathog..

[104] Grande R., Puca V., Muraro R. (2020). Antibiotic Resistance and Bacterial Biofilm. Expert Opin. Ther. Pat..

[105] Kumar M.A., Baba S.K., Sadida H.Q., Marzooqi S.A., Jerobin J., Altemani F.H., Algehainy N., Alanazi M.A., Abou-Samra A.-B., Kumar R. (2024). Extracellular Vesicles as Tools and Targets in Therapy for Diseases. Signal Transduct. Target. Ther..

[106] Tarashi S., Zamani M.S., Omrani M.D., Fateh A., Moshiri A., Saedisomeolia A., Siadat S.D., Kubow S. (2022). Commensal and Pathogenic Bacterial-Derived Extracellular Vesicles in Host-Bacterial and Interbacterial Dialogues: Two Sides of the Same Coin. J. Immunol. Res..

[107] Mohammadipoor A., Hershfield M.R., Linsenbardt H.R., Smith J., Mack J., Natesan S., Averitt D.L., Stark T.R., Sosanya N.M. (2023). Biological Function of Extracellular Vesicles (EVs): A Review of the Field. Mol. Biol. Rep..

[108] Muskan M., Abeysinghe P., Cecchin R., Branscome H., Morris K.V., Kashanchi F. (2024). Therapeutic Potential of RNA-Enriched Extracellular Vesicles: The next Generation in RNA Delivery via Biogenic Nanoparticles. Mol. Ther..

[109] Ren L., Zhang D., Pang L., Liu S. (2024). Extracellular Vesicles for Cancer Therapy: Potential, Progress, and Clinical Challenges. Front. Bioeng. Biotechnol..

[110] Du S., Guan Y., Xie A., Yan Z., Gao S., Li W., Rao L., Chen X., Chen T. (2023). Extracellular Vesicles: A Rising Star for Therapeutics and Drug Delivery. J. Nanobiotechnol..

[111] Zeng M., Liu M., Tao X., Yin X., Shen C., Wang X. (2024). Emerging Trends in the Application of Extracellular Vesicles as Novel Oral Delivery Vehicles for Therapeutics in Inflammatory Diseases. Int. J. Nanomed..

[112] Ming-Kun C., Zi-Xian C., Mao-Ping C., Hong C., Zhuang-Fei C., Shan-Chao Z. (2023). Engineered Extracellular Vesicles: A New Approach for Targeted Therapy of Tumors and Overcoming Drug Resistance. Cancer Commun..

[113] Cheng K., Kalluri R. (2023). Guidelines for Clinical Translation and Commercialization of Extracellular Vesicles and Exosomes Based Therapeutics. Extracell. Vesicle.

[114] Ahmed M., Kayode H., Okesanya O., Ukoaka B., Eshun G., Mourid M., Adigun O., Ogaya J., Mohamed Z., Lucero-Prisno D. (2024). CRISPR-Cas Systems in the Fight Against Antimicrobial Resistance: Current Status, Potentials, and Future Directions. Infect. Drug Resist..

[115] Kadkhoda H., Gholizadeh P., Samadi Kafil H., Ghotaslou R., Pirzadeh T., Ahangarzadeh Rezaee M., Nabizadeh E., Feizi H., Aghazadeh M. (2024). Role of CRISPR-Cas Systems and Anti-CRISPR Proteins in Bacterial Antibiotic Resistance. Heliyon.

[116] Ali N., Vora C., Mathuria A., Kataria N., Mani I., Singh V. (2024). Chapter Four—Advances in CRISPR-Cas Systems for Gut Microbiome. Progress in Molecular Biology and Translational Science.

[117] Vialetto E., Miele S., Goren M.G., Yu J., Yu Y., Collias D., Beamud B., Osbelt L., Lourenço M., Strowig T. (2024). Systematic Interrogation of CRISPR Antimicrobials in Klebsiella Pneumoniae Reveals Nuclease-, Guide- and Strain-Dependent Features Influencing Antimicrobial Activity. Nucleic Acids Res..

[118] Wei J., Li Y. (2023). CRISPR-Based Gene Editing Technology and Its Application in Microbial Engineering. Eng. Microbiol..

[119] Sen D., Mukhopadhyay P. (2024). Antimicrobial Resistance (AMR) Management Using CRISPR-Cas Based Genome Editing. Gene Genome Ed..

[120] León-Buitimea A., Garza-Cárdenas C.R., Román-García M.F., Ramírez-Díaz C.A., Ulloa-Ramírez M., Morones-Ramírez J.R. (2022). Nanomaterials-Based Combinatorial Therapy as a Strategy to Combat Antibiotic Resistance. Antibiotics.

[121] Liu Y., Qin R., Zaat S., Breukink E., Heger M. (2015). Antibacterial Photodynamic Therapy: Overview of a Promising Approach to Fight Antibiotic-Resistant Bacterial Infections. J. Clin. Transl. Res..

[122] Correia J.H., Rodrigues J.A., Pimenta S., Dong T., Yang Z. (2021). Photodynamic Therapy Review: Principles, Photosensitizers, Applications, and Future Directions. Pharmaceutics.

[123] Li D., Liu P., Tan Y., Zhang Z., Kang M., Wang D., Tang B.Z. (2022). Type I Photosensitizers Based on Aggregation-Induced Emission: A Rising Star in Photodynamic Therapy. Biosensors.

[124] El-Gendy A., Ezzat S., Abdel Samad F., Dabbous O., Dahm J., Hamblin M., Mohamed T. (2024). Studying the Viability and Growth Kinetics of Vancomycin-Resistant Enterococcus Faecalis V583 Following Femtosecond Laser Irradiation (420–465 Nm). Lasers Med. Sci..

[125] Salmani-Zarchi H., Mousavi-Sagharchi S., Sepahdoost N., Ranjbar-Jamalabadi M., Gross J., Jooya H., Samadi A. (2024). Antimicrobial Feature of Nanoparticles in the Antibiotic Resistance Era: From Mechanism to Application. Adv. Biomed. Res..

[126] Puri A., Mohite P., Maitra S., Subramaniyan V., Kumarasamy V., Uti D.E., Sayed A.A., El-Demerdash F.M., Algahtani M., El-kott A.F. (2024). From Nature to Nanotechnology: The Interplay of Traditional Medicine, Green Chemistry, and Biogenic Metallic Phytonanoparticles in Modern Healthcare Innovation and Sustainability. Biomed. Pharmacother..

[127] Ferraz M.P. (2024). Advanced Nanotechnological Approaches for Biofilm Prevention and Control. Appl. Sci..

[128] Modi S., Inwati G.K., Gacem A., Saquib Abullais S., Prajapati R., Yadav V.K., Syed R., Alqahtani M.S., Yadav K.K., Islam S. (2022). Nanostructured Antibiotics and Their Emerging Medicinal Applications: An Overview of Nanoantibiotics. Antibiotics.

[129] Johnson K.B., Wei W., Weeraratne D., Frisse M.E., Misulis K., Rhee K., Zhao J., Snowdon J.L. (2021). Precision Medicine, AI, and the Future of Personalized Health Care. Clin. Transl. Sci..

[130] Fernandes R. (2025). The Convergence of Nanotechnology and Biotechnology in Modern Medicine. Nanomaterials.

[131] Strathdee S.A., Hatfull G.F., Mutalik V.K., Schooley R.T. (2023). Phage Therapy: From Biological Mechanisms to Future Directions. Cell.

[132] Solanki R., Makwana N., Kumar R., Joshi M., Patel A., Bhatia D., Sahoo D.K. (2024). Nanomedicines as a Cutting-Edge Solution to Combat Antimicrobial Resistance. RSC Adv..

[133] Ioannou P., Baliou S., Samonis G. (2024). Nanotechnology in the Diagnosis and Treatment of Antibiotic-Resistant Infections. Antibiotics.

[134] Paul S., Verma S., Chen Y.-C. (2024). Peptide Dendrimer-Based Antibacterial Agents: Synthesis and Applications. ACS Infect. Dis..

[135] Benyamini P. (2024). Beyond Antibiotics: What the Future Holds. Antibiotics.

[136] Hariram N.P., Mekha K.B., Suganthan V., Sudhakar K. (2023). Sustainalism: An Integrated Socio-Economic-Environmental Model to Address Sustainable Development and Sustainability. Sustainability.

